# Randomized cross-over evaluation of investigator gender on pain thresholds in healthy volunteers

**DOI:** 10.3205/000301

**Published:** 2021-11-29

**Authors:** Anna Sellgren Engskov, Ilja Lejbman, Jonas Åkeson

**Affiliations:** 1 Department of Clinical Sciences Malmö, Anaesthesiology and Intensive Care Medicine, Lund University, Malmö, Sweden

**Keywords:** experimenter, gender identity, humans, pain measurement, sex, visual analog scale

## Abstract

**Background and aims:** This randomized cross-over study in healthy volunteers was designed primarily to evaluate the potential impact of investigator gender on electrical pain threshold (EPT) and corresponding pain intensity levels, and secondly to evaluate potential differences in those interventions between female and male study participants.

**Methods:** Forty adult volunteers (22 females) were included. An electrical stimulation device was used to determine EPT levels (in pain magnitude scores) in series of three in each study participant – once by a female, and once by a male investigator – according to a predefined cross-over design schedule. Corresponding levels of pain intensity were scored on a visual analog scale (VAS) slide ruler.

**Results:** Study data was obtained and analysed in all participants. Significantly higher EPT levels were determined by the female investigator compared with the male investigator (median 22 (IQR 12–31) vs. 8 (6–10) pain magnitude scores; *p*<0.0001), despite similar levels of reported pain intensity (1.9 (1.2–3.0) vs. 2.0 (1.1–3.4) VAS units; *p*>0.300). There were no differences in EPT levels between female and male subjects evaluated by female (*p*>0.300) and male (*p*=0.125) investigators, or between the first and second series of stimulation (*p*>0.300).

**Conclusions:** Our finding of significantly higher EPT levels when study participants of both genders – despite no difference in reported pain intensity – were evaluated by a female than by a male investigator, indicates a potential impact of investigator gender on the individual perception of pain.

**Implications:** By contributing to a better understanding of how individual pain threshold levels are potentially influenced by investigator gender, this study might facilitate future evaluation of pain conditions in both preclinical and clinical settings.

## Zusammenfassung

**Hintergrund und Ziele:** Diese präklinische randomisierte Cross-Over-Studie wurde entwickelt, um 1) einen möglichen Einfluss des Geschlechts der Untersucher auf die Schwellenwerte für elektrische Schmerzen (EPT) und entsprechende Schmerzintensität bei gesunden Freiwilligen zu bewerten, und 2) mögliche Unterschiede bei diesen Interventionen zwischen weiblichen und männlichen Studienteilnehmern zu bewerten.

**Methoden:** Vierzig gesunde erwachsene Freiwillige (22 Frauen) wurden eingeschlossen. Mit einem elektrischen Stimulationsgerät wurden die EPT-Spiegel in Dreierreihen bei jedem Studienteilnehmer – einmal von einem weiblichen und einmal von einem männlichen Untersucher – gemäß einem vordefinierten Cross-Over-Entwurfsplan bestimmt. Entsprechende Niveaus der Schmerzintensität wurden auf einem VAS-Lineal bewertet.

**Ergebnisse:** Studiendaten wurden bei allen Teilnehmern erhoben und analysiert. Im Vergleich zum männlichen Untersucher wurden von der weiblichen Untersucherin signifikant höhere EPT-Spiegel bestimmt (Median 22 (IQR 12–31) vs. 8 (6–10) Schmerzstärkeneinheiten; *p*<0,0001)), trotz ähnlicher Niveaus der berichteten Schmerzintensität (1,9 (1,2–3,0) vs. 2,0 (1,1–3,4) VAS-Einheiten; *p*>0,300). Es gab keine Unterschiede in EPT zwischen weiblichen und männlichen Teilnehmern, die von weiblichen (*p*>0,300) und männlichen (*p*=0,125) Untersuchern bewertet wurden, oder zwischen der ersten und zweiten Stimulationsreihe (*p*>0,300).

**Schlussfolgerungen:** Dass signifikant höhere EPT-Werte bei Studienteilnehmern beiden Geschlechts vorlagen, wenn sie von einer weiblichen statt einer männlichen Person untersucht wurden – trotz gleicher gemeldeter Schmerzintensität –, zeigt einen möglichen Einfluss des Geschlechts des Untersuchers auf die individuelle Wahrnehmung von Schmerz.

**Implikationen:** Durch diesen Beitrag zu einem besseren Verständnis, wie die individuellen EPT-Werte möglicherweise vom Geschlecht des Untersuchers beeinflusst werden, könnte diese Studie die zukünftige Bewertung von Schmerzzuständen sowohl im präklinischen als auch im klinischen Umfeld erleichtern.

## Introduction

Individual perception of pain is generally believed to be influenced by both physiological and psychosocial factors. The biological term ‘sex’ and the social term ‘gender’ (according to definitions by the World Health Organization) have both been proposed to be important regarding study participants [1] as well as investigators [2] in this context. To reflect how they are frequently being perceived, the term ‘gender’ is mainly used in this study.

Pain threshold is the level where extreme temperatures and pressures (or injury-related chemicals) activate nociceptors, i.e. peripheral sensory neurons, with following transduction and processing of stimuli in higher brain centres, resulting in pain perception [3].

Evaluations by female investigators have been associated with higher thresholds of pain induced by mechanical pressure [4] or laser [5] stimulation, and with lower intensity of pain induced by heat [6] or cold [7] stimulation in study participants. However, female investigators have also been reported to obtain similar levels of pain threshold, but lower levels of pain tolerance to cold-induced nociceptive stimulation [8]. Higher warm and cool thresholds, but not heat and cold pain thresholds, have been found in subjects evaluated by investigators of opposite gender [9]. The influence of investigator gender on pain perception after heat stimulation has been reported not to be associated with corresponding physiological changes in heart rate [6], possibly reflecting perceived traditional gender roles [4], [7], [9].

Numerous original studies on subject gender with different pain stimuli have found lower pain threshold levels [8], [10], [11], [12], [13], [14], [15], [16], [17], [18], [19], [20] or higher pain intensity levels [7], [21], [22], [23] in females – recently confirmed in two reviews [1], [24], possibly reflecting physiological sex differences [21] or psychosocial factors [25] like gender-role expectations [26], [27] – whereas others have found no differences [5], [9], [13], [27], [28].

Based on available data in females and males evaluated by investigators of both genders [4], [5], [7], [17], [23], our main study hypothesis was that evaluations by female investigators result in higher pain threshold levels and/or lower pain intensity levels than evaluations by male investigators. Our second study hypothesis was that females have lower pain threshold levels and/or report higher pain intensity levels than males regardless of investigator gender.

This randomized paired cross-over study was designed to evaluate potential impact of the gender of study investigators and participants on electrical pain threshold (EPT) levels and pain intensity levels.

## Subjects and methods

### Study setting

This prospective randomized paired cross-over study in adult healthy volunteers, approved by the regional Human Research Ethics Review Board (Approval No. 2015/779), Lund, and carried out in May 2016 at Skåne University Hospital, Malmö, Sweden, was designed to evaluate and compare EPT levels and individually scored pain intensity levels in study participants of both genders.

 Each participant was evaluated twice, at 10-to-15-minute intervals during daytime, according to a predefined randomized cross-over design schedule, by a 36-year-old female and a 27-year-old male resident in anaesthesiology and intensive care medicine with similar BMI and external appearance. The investigators, dressed in white physician coats, provided identical study information by reading a defined text from a paper in a private and quiet room. They knew the main purpose of the study.

### Subjects

Forty healthy adult volunteers (22 females) with normal health declaration and no current history of pain, use of analgesics, or use of other drugs affecting pain perception, were included after normal physical examination and individual verbal and written informed consent. Extensive physical activity within twelve hours and use of analgesic or alcohol within 24 hours before the study sessions were not allowed. All subjects were informed in advance by e-mail that their pain thresholds were to be determined twice with an established device designed for that specific purpose, but no instructions were given on how to use it. They were not informed about the main purpose of the study – evaluating potential influence of investigator and participant gender on pain perception – until after having participated.

### Induction of pain

In each study participant, pain was then induced in two series (at least ten minutes apart) of three stimulations with an electrical stimulation device (Painmatcher^®^, Cefar Medical AB, Lund, Sweden), delivering rectangular electrical pulses of 10 Hz frequency and 15 mA amplitude. Each subject was told to close an electrical circuit – to gradually increase the pulse duration stepwise by 4 µs – by pressing two buttons on the device between their right thumb and index finger pulps, and then to release the buttons as soon as the level of stimulation was considered to be painful, i.e. defined to correspond to their individual EPT, measured in pain magnitude scores (0–99), on a hidden display. The design of the device makes it involve the same parts of the fingers during all stimulations in each individual. Two identical devices, calibrated immediately before the study period by the Department of Medical Techniques, Skåne University Hospital, Malmö, Sweden, were used. Each device was used by each investigator in half of the study participants.

### Evaluation of pain

Immediately after each series of three stimulations, the maximum intensity at pain threshold was scored by the participant, according to established principles, on a horizontal visual analog scale (VAS) slide ruler, and subsequently handed over for the investigator to record the score from the back side with one decimal.

### Statistics

The influence of investigator gender on individually reported levels of pain intensity has previously been confirmed in 64 volunteer participants, subjected to experimental thermal pain and evaluated by VAS scoring with an unpaired study design [6]. Based on those findings, 40 study participants were estimated to be enough for evaluation of electrical pain thresholds in the present study, taking up to 20 percent potential dropouts into account. This number of participants was calculated to enable a difference of 6±12 pain magnitude scores between evaluations by female and male investigators to be confirmed with 80% statistical power and 95% statistical probability, based on paired cross-over comparison.

Individual EPT levels were calculated as average values of the three EPT values recorded in each series of stimulation. The Wilcoxon signed rank test was used to compare EPT levels and corresponding pain intensity score levels in study participants evaluated by female and male investigators, and to evaluate potential order and carry-over effects by comparing pain responses to the first and second series of stimulation. The Mann-Whitney U-test was used to compare EPT levels and pain intensity score levels between female and male subjects.

Parametric data is reported as mean ± standard deviation (SD), and non-parametric data as median with interquartile range (IQR) in parenthesis.

Levels of probability (*p*) below 0.01 were considered to reflect statistical significance to enable up to five multiple tests.

## Results

### Subjects

Results were obtained and analysed in 40 (22 female) 26±4-year-old subjects with a body weight of 68±11 kg and a body height of 175±10 cm.

### Induction of pain 

Each study participant followed the verbal instructions carefully and perceived the pain task accordingly, once with the female and once with the male investigator.

**Figure 1:**
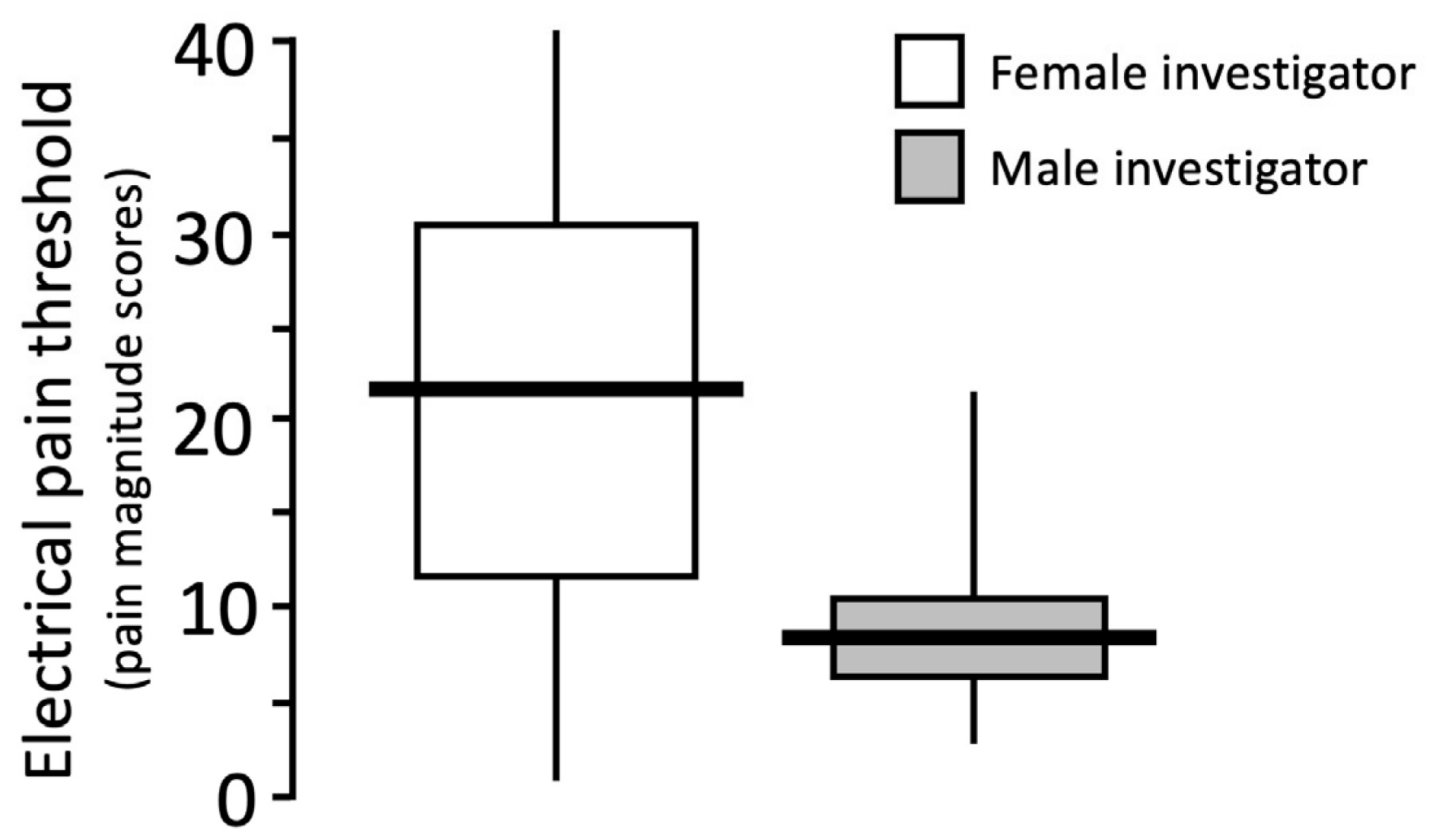
Electrical pain thresholds determined in 40 (22 female) healthy volunteers, evaluated by female and male investigators according to a randomized paired cross-over study design. Median values are indicated by bold horizontal lines, interquartile ranges by boxes, and ranges by vertical lines.

Individually calculated EPT levels were significantly higher (*p*<0.0001) when obtained by the female (median 22 (IQR 12–31) pain magnitude scores) than by the male (8 (6–10) pain magnitude scores) investigator (Figure 1 (Fig. 1)), and were higher in 33 evaluations (82%) made by the female. The EPT levels did not differ between female and male subjects evaluated by female (*p*>0.300) or male (*p*=0.125) investigators (Table 1 (Tab. 1)), or between the first and second series of stimulation (11 (7–19) vs. 11 (6–22) pain magnitude scores; *p*>0.300).

### Evaluation of pain 

**Figure 2:**
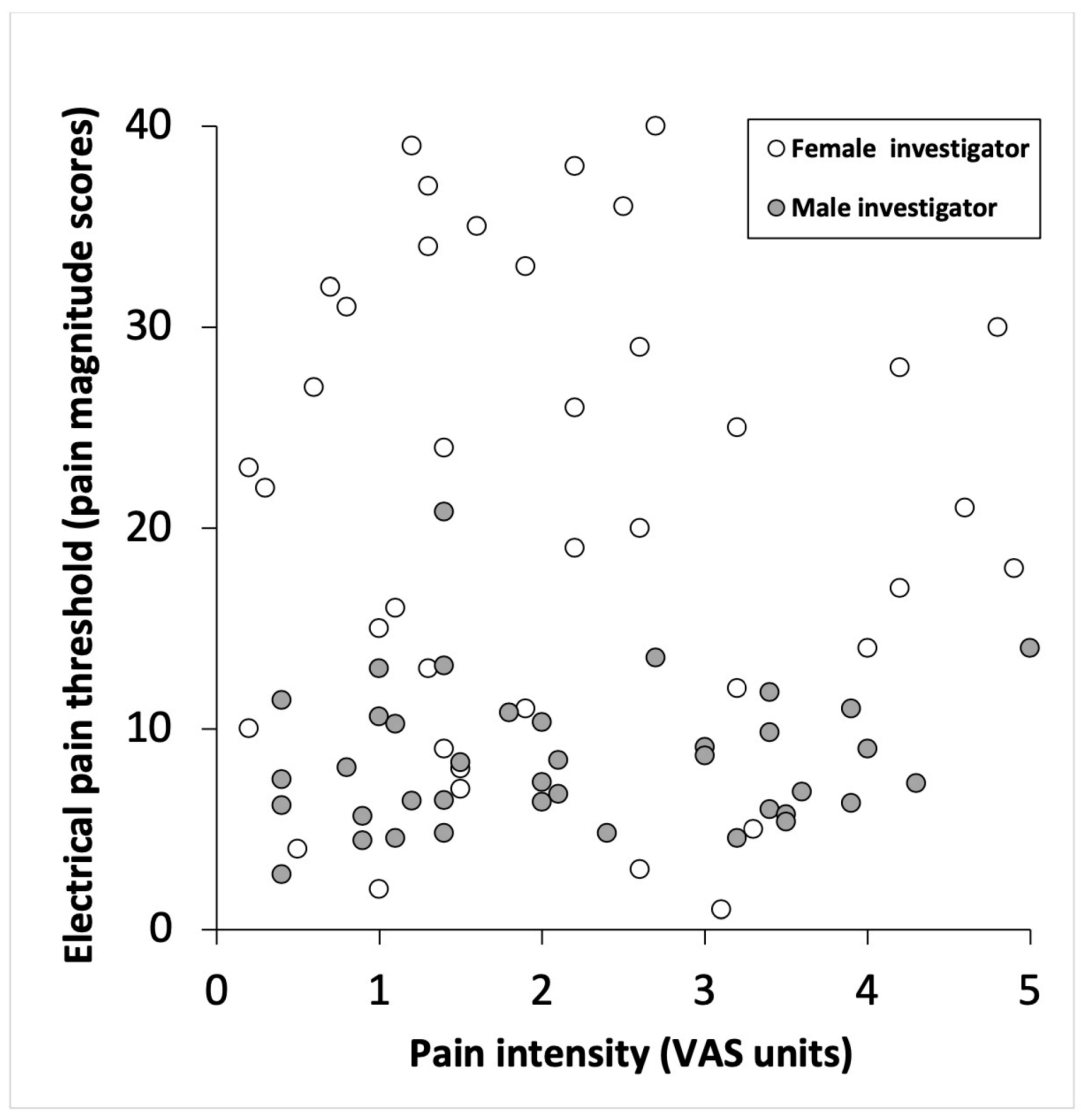
Individual electrical pain thresholds and corresponding visual analog scale (VAS) assessments of pain intensity in 40 (22 female) healthy volunteers, evaluated by female and male investigators according to a randomized paired cross-over study design

As shown in Table 2 (Tab. 2) and Figure 2 (Fig. 2), despite different EPT levels, pain intensity scores obtained by the female (1.9 (1.2–3.0) VAS units) did not differ (*p*>0.300) from those obtained by the male (2.0 (1.1–3.4) VAS units), or between female and male subjects evaluated by the female or male investigators (*p*>0.300). Higher (*p*>0.300) pain intensity scores were reported during the first (2.1 (1.1–3.2) VAS units) than during the second (1.8 (1.2–3.1) VAS units) series of stimulation.

## Discussion

This is the first study to confirm an influence of investigator gender on pain perception in response to electrical pain stimulation, as far as we know. Our findings of higher EPT levels in females and males investigated by a female than by a male are in partial agreement with results obtained in males in the early 1990s by cold (with lower pain intensity) [7] and in the mid-2000s by mechanical pressure (with higher pain threshold) [4]. However, since those investigators were dressed to emphasize their gender roles, those results were interpreted to primarily reflect influence of traditional gender role expectations, as also proposed in a survey of epidemiological and laboratory data on sex differences in pain perception [29]. Nevertheless, another study from the mid-2000s and a recent study, both based on heat-induced pain, not emphasizing traditional investigator gender roles in line with our study, have also reported higher pain thresholds in males evaluated by a neutrally dressed female [5], [6], representing more realistic clinical patient-physician interaction.

Furthermore, males subjected to heat reported lower pain intensity levels when investigated by a female than by a male [6], but with no corresponding change in heart rate, possibly reflecting a primarily psychosocial influence on pain perception.

Intimate study settings, with a calm and quiet one-to-one environment, have been reported to facilitate non-verbal patient-physician interaction [10], and could hence be considered to accordingly promote communication between study participants and investigators. Female television spokespeople have recently been reported to communicate more extensively non-verbally [30], e.g. by more frequent smiling and eye contact [31], and a neurophysiological study [32] has reported stronger empathic abilities of females dealing with pain in others. Hence, the intimate study setting, potentially promoting non-verbal communication and empathic interaction, might have contributed to the main findings of the present study.

Our statistically non-differing EPT levels between female and male study participants are in accordance with diverging results reported in early studies based on electrical stimulation [33]. Reviews of later studies, also including other modalities of stimulation, support differences [24], [34], [35] as well as no differences [35] in pain perception between subject genders. No differences in pain perception were reported between female and male study participants in response to pain induced by cold [27], heat [9], [13], or venous cannulation [28]. In contrast, female subjects had lower threshold levels of pain induced by electricity [10], [13], [16], [36], mechanical pressure [11], [14], [18], [19], [37], heat [12], [14], [17], or cold [8], or reported higher intensity levels of pain induced by heat [21], [23] or cold [7], [22].

Factors underlying sex or gender differences in pain perception – physiological as well as psychosocial – are not yet fully understood [24], [34]. Higher nociceptive discrimination in females subjected to heat-induced pain [21] might indicate that gender differences in pain perception reflect physiological rather than psychosocial factors in agreement with a recent review [38] implying impact of humoral factors on central mediation of pain. Moreover, the perception of pressure-induced pain has been reported to be more influenced by sex than by gender [11], [37], and higher thermal-induced pain thresholds in males have been associated with higher activity of the parasympathetic nerve system [39]. However, cognitive and social factors have also been proposed to partly explain higher pain perception in females [25] and particularly reflect gender role expectations [26], [27], which is in agreement with higher pain thresholds found in subjects considering themselves more masculine according to a previous review [40]. Differences in pain perception between female and male subjects, not adjusted for investigator gender [11], [12], [13], [14], [17], [18], [19], [36], might actually, at least in part, also reflect a psychosocial impact of investigator gender [41].

Although we involved only one investigator of each gender to avoid interindividual variation, whereas up to four female and four male investigators have been involved in similarly designed but unpaired studies [6], [8], [37], this might be considered as a study limitation, despite similar external appearance, body size, generation and profession, and use of a predefined verbal script. The study investigators were not blinded to the main purpose of the study, in contrast to a similar previous study [7], which might also be a limitation. Parallel use of two identical and calibrated electrical devices (to enable blinding of the participants to the main study purpose) might also be considered as a limitation.

Advantages of this study are the blinding of participants to the main study purpose (to avoid some psychosocial impact on pain perception) and of both participants and investigators to on-line EPT levels. The use of a valid and reliable [42], [43] investigator-independent stimulation device designed for this specific purpose instead of investigator-dependent ones [4], [5], [6], [7] is considered to be another advantage. Combining EPT determination with individual VAS scoring – considered as the gold standard for pain assessment [44] – empowers our findings.

Carry-over effects were avoided by allowing sufficient time between the study sessions, and order effects by the randomized paired cross-over study design not used in previous similar studies [4], [6], [7], [9], [21], [22], [23], [45]. Furthermore, our main results are supported by statistical significance levels also taking multiple comparisons into consideration.

In conclusion, our main findings of higher EPT levels despite equal pain intensity scores in females and in males evaluated by a female investigator contribute to a better understanding of investigator gender impact on pain perception. Since these results may also have clinical relevance, future studies on investigator gender and pain perception in clinical settings are desirable, particularly considering the predominance of female staff in modern healthcare.

## Notes

### Informed consent

Informed consent has been obtained from all study participants.

### Ethical approval

This research in humans complies with all the relevant national regulations and institutional policies, was performed in accordance with the tenets of the Helsinki Declaration, and has been approved by the regional Human Research Ethics Review Board (Approval No. 2015/779), Lund, Sweden.

### Funding

The study was supported by research grants provided by Region Skåne, Kristianstad, and by research funds administered by Lund University Faculty of Medicine, Lund, Sweden.

### Competing interests

The authors declare that they have no competing interests.

## Figures and Tables

**Table 1:**
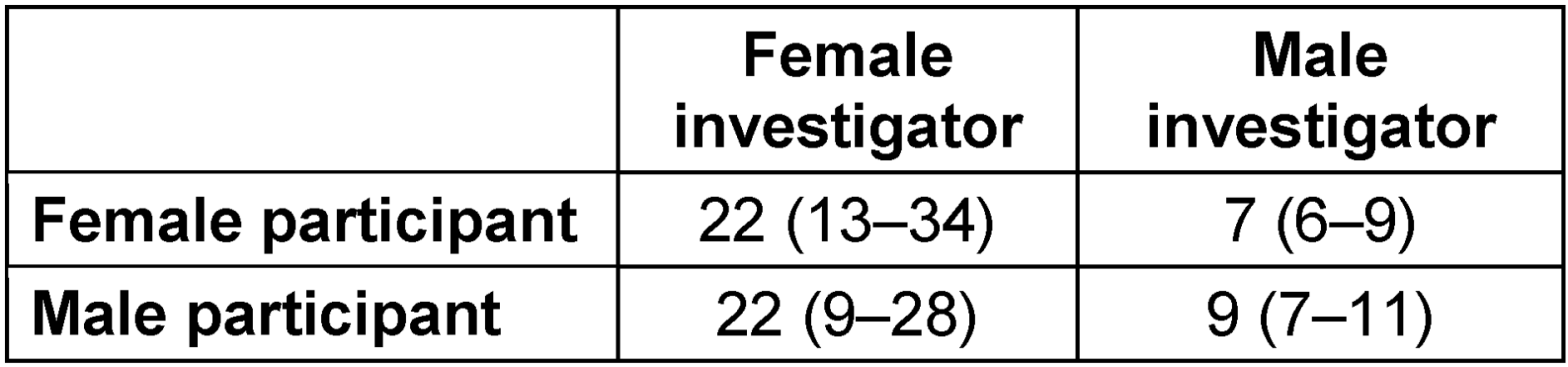
Electrical pain threshold levels (median values with interquartile ranges) in 40 (22 female) healthy volunteers, evaluated by female and male investigators according to a randomized paired cross-over study design

**Table 2:**
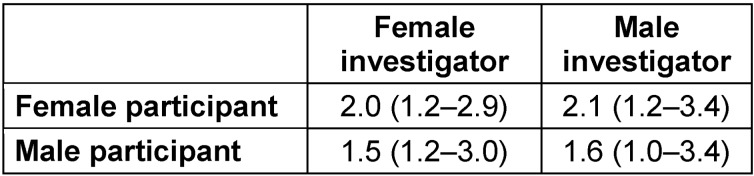
Pain intensity levels (median values with interquartile ranges) in 40 (22 female) healthy volunteers, evaluated by female and male investigators according to a randomized paired cross-over study design
